# Testing of a novel automated point-of-care analyzer for blood ammonium monitoring in a clinical setting

**DOI:** 10.1007/s00216-025-05879-z

**Published:** 2025-04-23

**Authors:** Beatriz Rebollo-Calderón, Antonio Calvo-López, Aida Ormazábal, Rafael Artuch, Javier Rosell-Ferrer, Julian Alonso-Chamarro, Mar Puyol

**Affiliations:** 1 Group of Sensors and Biosensors, Department of Chemistry, Autonomous University of Barcelona, Edifici Cn, Bellaterra, Barcelona, 08193 Spain; 2 Institut de Recerca Sant Joan de Déu, Passeig de Sant Joan de Déu, 2, Esplugues de Llobregat, Barcelona, 08950 Spain; 3 Centre de Recerca en Enginyeria Biomèdica (CREB), Universitat Politécnica de Catalunya (UPC), Campus Diagonal Sud, Edifici H., Av. Diagonal 647, Barcelona, Spain

**Keywords:** Potentiometry, Hyperammonemia, Inborn error of metabolism, Point-of-care, Ammonium selective electrodes

## Abstract

Certain diseases are marked by elevated ammonium levels in the blood, a condition known as hyperammonemia. Prompt detection and medical intervention are crucial to prevent potentially fatal outcomes. Therefore, ammonium levels should be monitored regularly, typically in referral hospitals where specialized and costly equipment is available. Although compact commercial devices are available for this purpose, none of them meet all the technical and analytical requirements needed for direct blood analysis, and current reported strategies have not been validated with enough samples to confirm results reliably. We present a robust and reliable automated point-of-care (POC) analyzer for the potentiometric determination of ammonium in blood. Comprising three computer-controlled modules—fluid management, detection, and data acquisition and transmission—this system combines portability, ease of use, and affordability. It can directly measure untreated blood samples, significantly reducing analysis time. Fully automated, it operates unsupervised with minimal lab personnel intervention. Analytical quality parameters include 5% RSD repeatability (n = 8), a limit of detection of 24 μM, a working range of 30–1000 µM and a sample volume of 215 µL. Successfully implemented in a hospital for 2 months, it analyzed 238 blood samples in parallel with the hospital’s reference method showing comparable results (paired *t*-test, Passing-Bablok regression and Bland–Altman Plot) and randomly distributed errors, with a 4% accuracy calculated as mean error. Results indicate the POC analyzer effectiveness and reliability in a clinical setting compared to currently reported or commercially available equipment, being suitable for bedside monitoring of conditions associated with hyperammonemia in healthcare centers, including emergency rooms and clinics in developing countries.

**Graphical Abstract:**

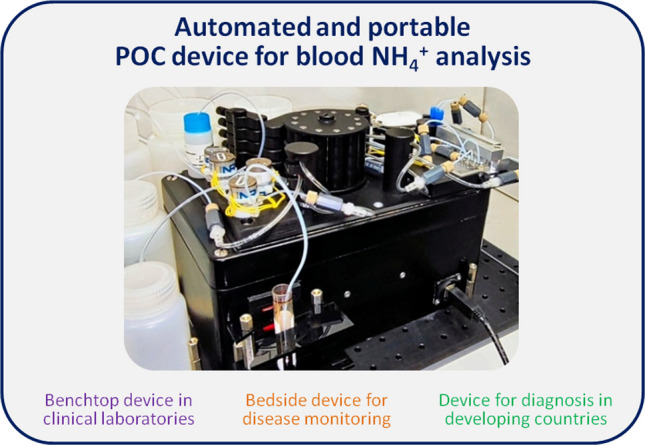

## Introduction

High levels of NH_4_^+^ in blood constitute a condition known as hyperammonemia, which is the main pathological trait of some inborn errors of metabolism (IEM). In particular, hyperammonemia is a common and severe complication of the urea cycle disorders (UCDs) and organic acidemias (OA), where ammonium elimination is impaired due to the disruption of the urea cycle due to the mutation of any of its enzymes or to reduced availability of some substrates [1]. Consequently, NH_4_^+^ increases over the healthy levels, which reach up to 50 μM in adults and children and up to 100 μM in newborns [2]. Concentrations higher than 200 μM constitute severe hyperammonemic states which may lead to poor neurological outcomes and permanent brain damage [3]. Even higher concentrations are related to hyperammonemic comma and death. Besides UCDs and OA, there are other non-congenital conditions that may cause hyperammonemia, either due to a decreased detoxification as in cirrhosis and hepatic failure or due to an increased production of the molecule as in the use of some drugs or in bacterial overgrowth [4, 5].

To avoid severe affectations to the neurological system it is imperative to detect and treat hyperammonemic episodes in a reliable and fast manner. Treatment is mainly focused in reducing NH_4_^+^ levels in blood by means of drugs, dialysis or hemofiltration [5]. Patients with UCDs and OA must also follow a low-protein diet in order to reduce protein catabolism [6].

Nowadays, the analysis of NH_4_^+^ levels in blood is carried out by means of enzymatic spectrophotometric analytical methods which are based on the reaction catalyzed by the enzyme glutamate dehydrogenase, using plasma as sample [7–9]. This analytical method allows reliable discrimination between healthy and pathological NH_4_^+^ levels. Nevertheless, it presents several drawbacks related to its high cost, large equipment dimensions, need of expert personnel and the requirement of a centrifugation step to obtain plasma, as whole blood is not a suitable sample. The latter issue is of particular importance, as blood samples need to be taken into the lab and treated, thus lengthening the overall time of the analysis. Besides delaying the access to the treatment, this step also presents a problem due to the quick increase of NH_4_^+^ in biological samples [10]. For all these reasons, NH_4_^+^ analysis is limited to laboratories of referral hospitals, hindering a fast detection of hyperammonemic states and a more efficient periodic biochemical monitoring.

In this sense, there is a need to develop reliable, low cost and easy to use analytical instrumentation, adaptable to health clinics and emergency rooms. Furthermore, low cost is of particular importance for a potential use in developing countries where these diseases cannot even be diagnosed. Likewise, portable equipment and direct blood analysis would permit bedside implementation as point-of-care (POC) analysis. Currently, various reported proposals are focused on the measurement of ammonium in blood as POC devices, including a commercial one (Table 1). However, they do not meet all the necessary requirements for bedside use. Some have working ranges that do not cover the entire pathological range (up to 1000 μM), others have very long analysis times, require sample pretreatment (such as centrifugation to obtain plasma, manual pH adjustment, or sample dilution), present interferences (mainly from the sample matrix), are poorly automated, exhibit a low technology readiness level (TRL), or despite showing promising potential have not been extensively clinically validated to ensure the reliability of their results.

**Table 1:** Review of different proposed devices for the detection of blood ammonium

Technique	Working range (μM)	Sample volume (μL)	Analysis time (min)	Sample pre-treatment	Interferences	Automation level	TRL	Clinical validation (n > 100 blood samples)	Ref
Colorimetry	7–286	20	4	No	No	Medium/high	High (commercial)	Yes	[9, 11]
Impedance spectroscopy	25–200	52	28	Yes	No	Medium	Low	No	[12]
Amperometry	4–1000	15	2	No	No	Medium	Medium	No	[13]
Colorimetry	28–556	200	15	Yes	Yes	Low	Low	No	[14]
Colorimetry	0.1–37	20	15	Yes	Yes	Medium	Low	No	[15]
Amperometry	0.05–256	20	1.6	Yes	No	Medium	Low	No	[15]
Potentiometry	30–1000	215	5*	No	No	High	Medium/high	Yes	This work

*At 150 μM

This work presents a reliable, fully automated point-of-care (POC) ammonium analyzer designed for direct blood monitoring of hyperammonemia. It is based on the use of ion selective electrodes (ISE) for the potentiometric determination of NH_4_^+^ and the use of a gas diffusion step to isolate the analyte from the sample matrix; an approach that has already been exploited in this type of microanalyzers [16–18]. The present full analytical system is an improved, automated and compact prototype derived from a previously reported proof-of-concept [19]. It consists of three different modules: *detection*, which in turn is composed of three units; *fluid management*; and *data acquisition and transmission*. The inclusion of the two latter modules conforms to a multicommutated flow analysis (MCFA) system. This way, different protocols for calibration procedure, control and sample analysis and system cleaning can be carried out in an automated and reproducible manner [20–22]. Additionally, MCFA can work autonomously or under minimum supervision by non-trained personnel.

The POC prototype was installed in the laboratory of the Hospital Sant Joan de Déu (HSJD) (Esplugues de Llobregat, Spain), referral hospital for NH_4_^+^ analysis, for its evaluation under continuous use for a 2-month period. During this time, 238 blood samples were analyzed in parallel with the reference method employed by the hospital for validation purposes.

## Materials and methods

### Materials

The detection module is composed of three different units for the following operations: mixing, gas diffusion and sensing. All three units were fabricated using the thermoplastic cyclic olefin co-polymer (COC). COC 8007 (of 25 μm thickness) and COC 6013 (of 400 μm thickness) were purchased from Tekni-Plex (Erembodegem, Belgium). The sensing unit included a reference electrode consisting of an Ag/AgCl paste (C2030812D3, Gwent, Pontypool, United Kingdom). It also included an ISE composed of a conductive support and an ion selective membrane. The former consisted of an epoxy-graphite resin constituted by 50% powdered graphite of 50 μm particle size (Merk, Germany), 36% epoxy Araldite-M (Ciba Geigy, Spain) and 14% hardener HR (Ciba Geigy, Spain). The latter was composed by 1% nonactin (Sigma-Aldrich, Spain), 33.5% PCV (Sigma-Aldrich, Spain) and 65.5% bis(1-butylpentyl)adipate (BBPA) (Sigma-Aldrich, Spain). Tetrahydrofuran (THF) (Sigma-Aldrich, Spain) was used as volatile solvent for the ion selective membrane.

To isolate NH_4_^+^ from the sample matrix, a hydrophobic polyvinylidene fluoride (PVDF) membrane of 125 μm thickness and 0.45 μm porous size purchased at Millipore was used. An additional membrane of polycarbonate (PC) with a porous size of 0.05 μm purchased at Whatman Nucleopore was used as a protective membrane to avoid direct contact between blood and the PVDF membrane.

Reagents used in this work included NH_4_Cl (Acros Organics, Belgium) for the preparation of the NH_4_^+^ stock solution, KCl (Sigma-Aldrich, Spain) to stabilize the potential at the reference electrode, NaOH (Sigma-Aldrich, Spain) and ethylenediaminetetraacetic acid (EDTA) (Panreac, Spain) as the donor solution and 2-[4-(2-hydroxyethyl)piperazin- 1-yl]ethanesulfonic acid (HEPES) (Sigma-Aldrich, Spain) as the acceptor buffer solution. Ba(OH)_2_ (Thermo Fisher Scientific, Spain) was used to adjust the pH of the buffer. All solutions were prepared using Milli-Q water. The control solution employed was the same as for the reference method (Ammonia Ultra Kit ABBOT, USA).

### POC design

**Fig. 1:**
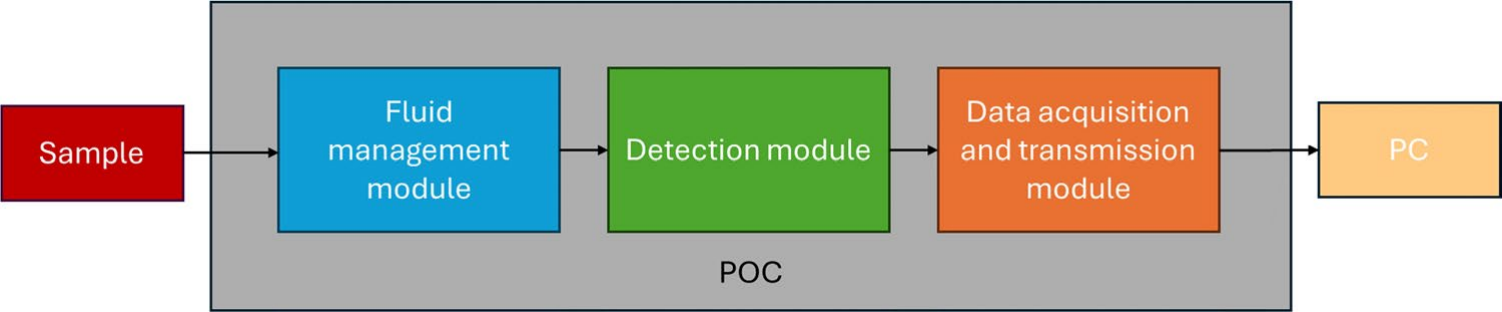
Schematic representation of the three modules that constitute the POC analytical system

As mentioned before, the analytical system is composed of three different computer-controlled modules: fluid management, detection, and data acquisition and transmission (Fig. 1).

The detection module consists of three different microfluidic units: (1) a micromixer where samples mix with a NaOH solution to transform NH_4_^+^ into volatile NH_3_; (2) a gas diffusion unit that contains the PVDF and the protective membrane; and (3) the sensing unit that includes the NH_4_^+^ ISE and the reference electrode. The design of the gas diffusion unit is one of the most critical parts of the detection module, and its original design from the previously published proof-of-concept has been modified to reduce pressure on the membranes surface and thus improving lifetime of the device.

The fluid management unit contains all the elements that are responsible for fluid handling, such as pumps, valves and control software. Data acquisition and data communication unit is composed by a potentiometer and a software that transfers data via Bluetooth, processes data and shows result on the display.

The compact POC dimensions (L x W x H) are 30 × 16 × 18 cm, and it weighs approximately 2 kg, which is adequate for its portability.

### Detection module

The fabrication technology of the different units that make up the detection module has been described in a previous publication [23]. The process is based on a layer-by-layer approach, alternating COC layers of different glass transition temperatures (T_g_). This approach allows the use of the COC layer with a higher Tg as structural layers and those with a lower Tg as sealing layers. Patterns of structural layers are designed by a computer-aided design (CAD) software and transferred on top of the thermoplastic by micromilling using a computer numerically controlled (CNC) micromilling machine (Protomat C100/HF, LPKF, Spain). Then, the conductive support is placed and cured overnight at 40 °C followed by a polishing process. Afterwards, the ion selective membrane is drop-casted on the detection chamber over the conductive support. Finally, a thermo-compression press (Francisco Camps, Granollers, Spain) is used to seal all the COC layers together (T = 102 °C and P = 4 bar). Prior to use, the ISEs must undergo a conditioning step by submersion into a 0.1 M NH_4_Cl solution for 4 h and into a 10^−4^ M NH_4_Cl solution for 16 h.

**Fig. 2:**
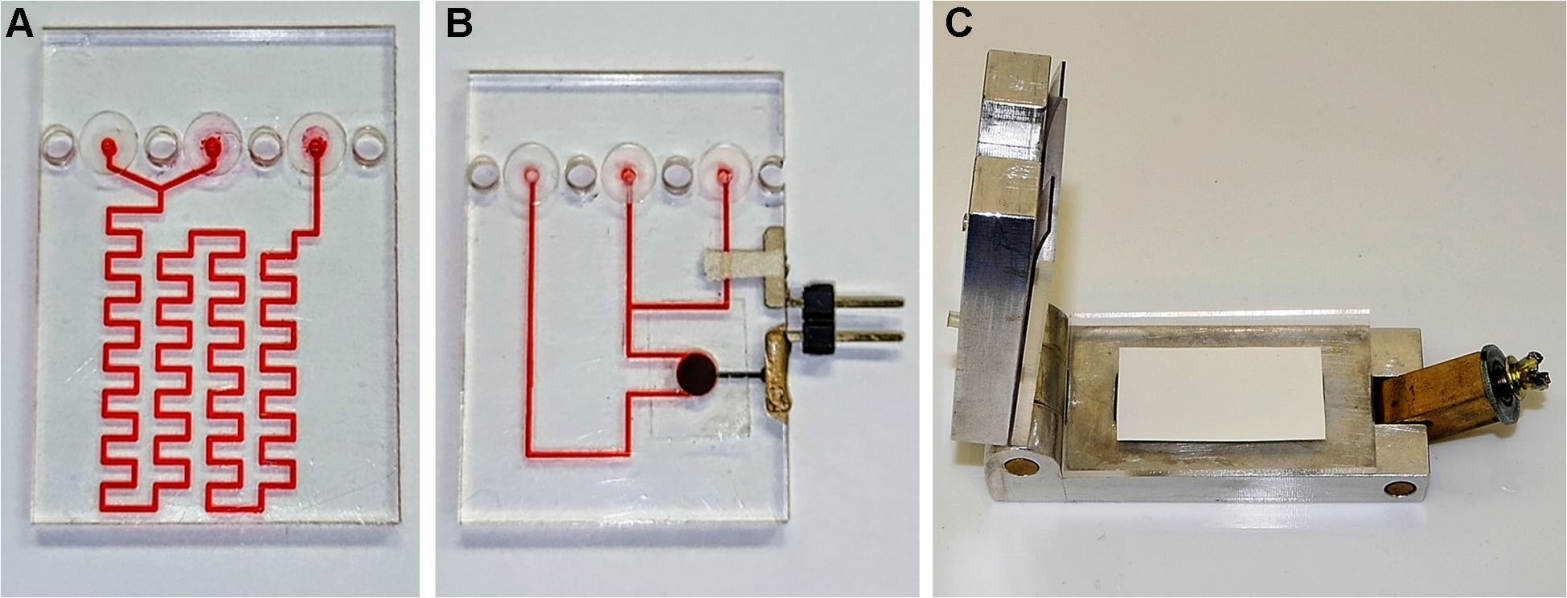
Real images of the different units that compose the detection module being: **A** the micromixing unit, **B** the sensing unit and **C** the gas diffusion unit in open position for membrane exchange

The three different units of the detection module have been fabricated individually to facilitate the optimization of the analyzer and allow the replacement of one of the components, if necessary, as each unit has a different service lifetime. This cuts costs and reduces the time required for the fabrication. Real images of the three units can be seen in Fig. 2.

### Fluid management, and signal acquisition and transmission modules

The fluid management unit includes a 4-channel peristaltic pump (Spetec, Germany), five 3-way injection valves (NResearch, NJ, USA) and a bubble trap (Elveflow, France). For the liquid propulsion, Tygon tubes of 1.14 mm and 0.64 mm internal diameter (Ismatec, Wertheim, Germany) and Teflon tubes of 0.8 mm internal diameter were used.

**Fig. 3:**
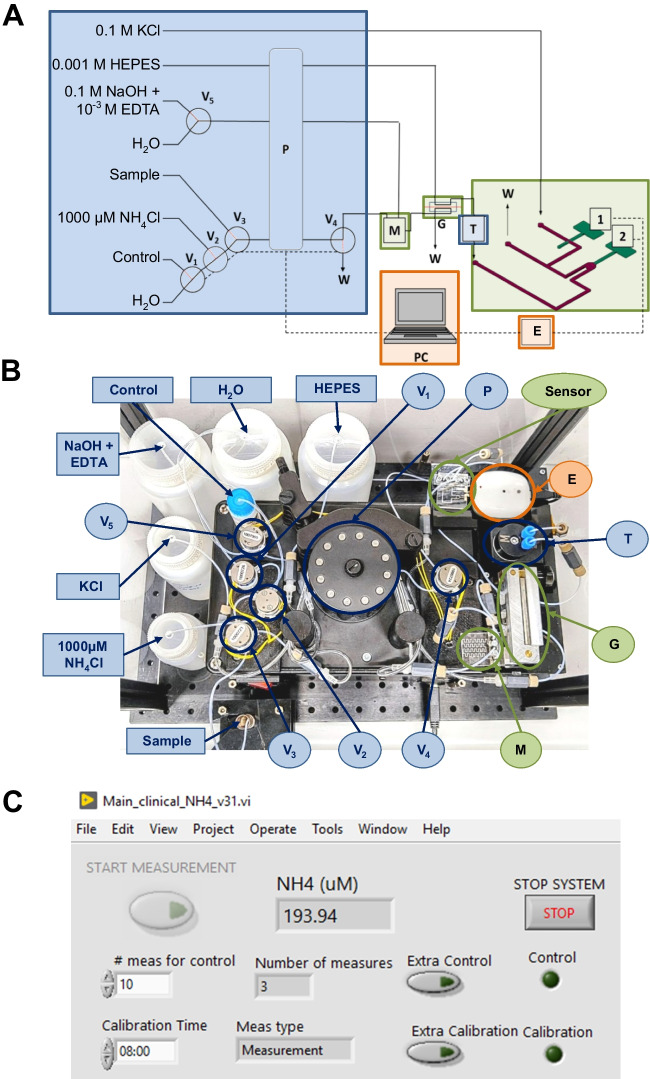
**A** Schematic representation of the automated experimental setup, with the fluid management module (blue), detection module (green) and the data acquisition and transmission module (orange) and **B** real image of the top view of the POC device were 1: reference electrode, 2: indicator electrode, E: Potentiometer, T: bubble trap, V_x_: 3-way injection valve, P: peristaltic pump, M: micromixer, G: gas diffusion unit, PC: computer, W: waste. **C** Screenshot of the interface of the developed Labview program, where there is the “start measurement” button to start the sample analysis, the ammonium concentration display to show the ammonium blood level, and there are other auxiliary buttons to stop the POC device and to perform an extra control analysis or calibration procedure, depending on the situation

The signal acquisition and transmission module includes a custom made miniaturized potentiometer developed by the Research Centre for Biomedical Engineering (CREB) of *Universitat Politècnica de Catalunya* (UPC). Data transmission is via Bluetooth. A Labview program, also developed by the CREB, manages both modules: it controls all the fluid management elements of the system and handles signal acquisition and data processing. A schematic representation of the full setup can be seen in Fig. 3A, alongside real images of the top view (Fig. 3B) of the POC*)* and a screenshot of the developed Labview program (Fig. 3C).

### Blood samples

The automated point-of-care (POC) system was installed in the HSJD laboratory for a 2-month continuous evaluation. During this period, 238 anonymized blood samples for routine ammonium determinations were analyzed concurrently using the novel POC system and the reference method. A 500 µL aliquot was collected anonymously, ensuring no sensitive patient information or identities were stored. The sampling batch included both spiked and non-spiked samples to cover the full expected physiological and pathological range of NH_4_^+^ in blood. All experiments were conducted within the HSJD laboratory, and no data was transferred to external hospital data repositories.

Blood samples were collected in tubes containing EDTA and analyzed in parallel. First, blood samples were analyzed by the POC; then, plasma fractions of these samples were analyzed by the reference method employed at the HSJD. For this purpose, plasma fractions were obtained after the centrifugation of blood samples for 10 min at 3000 rpm at 10 °C. The reference method consisted of an enzymatic method using an automated spectrophotometric procedure in an Architect ci8200 automated analyzer (ABBOT, Park, IL, USA) [8]. The reference method is accredited by ENAC agency following the ISO 15189 norm, and it is subjected to external and internal quality control schemes. These data are available on request.

## Results and discussion

### Detection module configuration

**Fig. 4:**
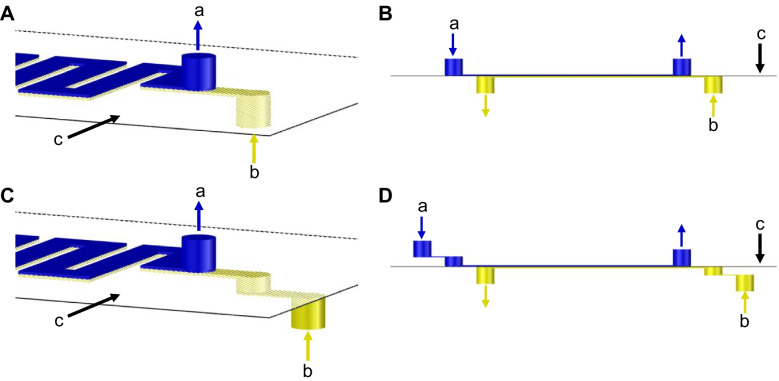
3D and 2D schematic representation of the lateral view for the original (**A** and **B**) and modified (**C** and **D**) configurations of the gas diffusion unit, showing the inlets and outlets of the donor (“a” or blue color) and the acceptor (“b” or yellow color) microchannels and the protective membrane and the gas diffusion membrane (c). The figure is not to scale

The configuration of the detection module is of particular interest, specifically, of the gas diffusion unit. In previous works [19], this module contained non-superposed straight-like inlets that lead to 100 µm deep microchannels (Fig. 4A). When using this configuration with the automated analytical system the protective and PDVF membranes were damaged on the inlet point of the donor side (which contains the NaOH solution and the sample) just after one injection of a blood sample. This was related to a high overpressure in this area. To solve this, inlets were modified to a step-like structure and the depth of microchannels was increased to 200 μm (Fig. 4C). These changes allowed the use of the same pair of membranes for 14 days and for the analysis of more than 100 blood samples.

Figure 4 3D and 2D schematic representation of the lateral view for the original (A and B) and modified (C and D) configurations of the gas diffusion unit, showing the inlets and outlets of the donor (“a” or blue color) and the acceptor (“b” or yellow color) microchannels and the protective membrane and the gas diffusion membrane (c). The figure is not to scale.

### Automation of the fluid management module

**Fig. 5:**
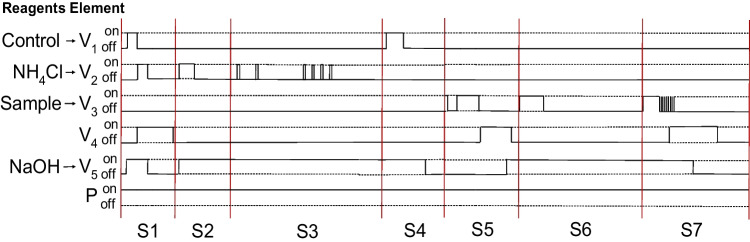
Schematic representation of the ON/OFF state for the different elements of the automated setup for steps 1 (S1), 2 (S2), 3 (S3), 4 (S4), 5 (S5), 6 (S6) and 7 (S7). (V) 3-way injection valve and (P) peristaltic pump

This analytical system was designed to perform all necessary steps for a complete analytical process in an automated manner. Personnel are only required to place and remove sample tube and cleaning solution. Several protocols constitute the total analysis process, which is accomplished through the multicommutation of various solenoid microvalves between their ON and OFF positions (Fig. 5). Prior to the first use, an automated priming process takes place to fill all the microfluidic tubes with the corresponding reagent (Step 1). Then, every day, prior to the calibration procedure, there is an injection of a 1000 μM NH_4_Cl stock solution to keep the ISE well-conditioned (Step 2). Afterwards, the calibration procedure is done by a single analysis of two NH_4_Cl standard solutions (50 μM and 100 μM) prepared by the automated dilution of the stock solution (Step 3). Next, a control solution (the same used for the Architect ci8200 automated analyzer) is analyzed to correct the previously obtained calibration curve and allow a proper comparison of both analytical methods (Step 4). Finally, blood samples are loaded (step 5) and analyzed (Step 6), and after each analysis a cleaning protocol takes place (Step 7). Control solution is also used to ensure that signal heights are kept stable and that there is no need to re-calibrate the system. When no analyses are taking place, the flow rate is reduced from 650 μL·min^−1^ to 70 μL·min^−1^ to minimize reagent consumption.

As can be seen in Fig. 3A, the actuation of different microvalves (valves 1 (V1), 2 (V2) and 3 (V3)) allows the injection of the control solution, the multicommutation protocol to carry out the calibration and the injection of the sample respectively. It is important to note that in MCFIA the injection volume is defined by the time of injection. Therefore, to obtain reliable results, it is crucial to ensure that all valves operate identically, injecting the same volume for a given injection time without deteriorating from continuous use. If any discrepancies arise, a correction must be applied. The repeatability of V1, V2 and V3 was assessed by 10 consecutive injections of a hand-prepared 100 μM NH_4_Cl solution obtaining in all cases relative standard deviations (RSD) values lower than 3%. Likewise, the state of V2 and its repeatability regarding the multicommutation sequences are important in order to achieve a reliable calibration curve. In this sense, 10 injections of two 100 μM NH_4_Cl solutions, obtained by hand-preparation and by multicommutation, reveal RSD values of 1% and 3% respectively, so the use of multicommutation involves a certain degree of acceptable variability.

For a one day of use, which includes an injection of the NH_4_^+^ stock solution, a calibration procedure, an initial control analysis, 20-sample analysis with the corresponding cleaning protocols and a control analysis every 10-sample analysis, we calculated the reagents consumption. Approximately, 113 mL of 0.1 M KCl, and 273 mL of water, 0.1 M NaOH and 0.001 M HEPES are consumed every day, and a total of 930 mL of waste is generated. Considering an initial volume of 1 L for solutions stored on-board, KCl must be replaced every 8 days and the rest of solutions every 3 days. Daily consumption of the NH_4_^+^ stock solution is negligible.

Regarding the lifetime of the different consumable elements of the POC equipment, the sensor unit has been set at 28 days and the gas diffusion unit at 14 days, while Tygon tubes must be changed every 60 days to ensure a stable flow rate. Lifespan of each component has been evaluated under intensive use of the equipment with the analysis of blood samples.

### Analytical characteristics of the POC system

The hydrodynamic parameters used for this POC system were a flow rate of 650 μL· min^−1^ and a time of injection of 20 s, which equals to an injection volume of 215 μL. Under these conditions, a calibration curve is obtained every day by a single analysis of two concentrations, 50 μM and 100 μM, of NH_4_Cl, generated by the multicommutated dilution of the stock solution. As an example, the Nernst equation obtained is of E = 57.8 · log [NH_4_^+^] + 273.1. This calibration curve is later corrected using the analysis of the same control solution than the reference method, generating a new equation of the same slope but different Y-intercept. For instance, E = 57.8 · log [NH_4_^+^] + 266.8. This way, the limit of detection for this corrected calibration curve is recalculated (in this example it would be 24.2 μM). Note that every day a new calibration is performed with 50–100 μM standards to achieve the highest results reliability in this range, where blood ammonium levels are considered altered in adulthood and in children, respectively. In any case, the equipment shows a linear range of 30 to 1000 μM verified in the previous characterization of the electrodes used in this POC device prototype [19].

The repeatability of the developed automated analytical system was evaluated by 8 consecutive analyses of a blood sample and 10 consecutive analyses of the control solution, obtaining acceptable RSD values of 5% and 3% respectively.

### Validation with blood samples

**Fig. 6:**
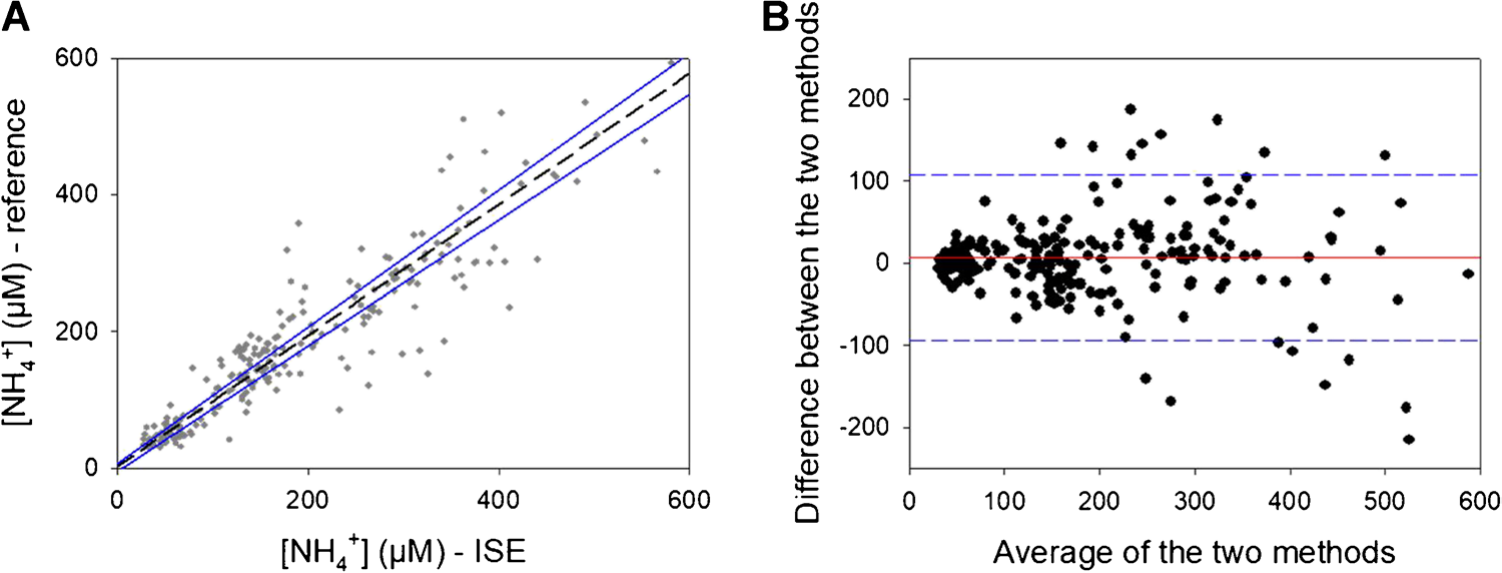
**A** Passing-Bablok regression (dashed line) with the CI (95%) indicated in blue. **B** Bland–Altman plot with the mean difference indicated in red and the limits of agreement (95%) indicated in blue. N = 238

It is important to highlight that the developed POC system analyzes blood samples, whereas the reference method is limited to plasma samples. Therefore, this validation involves two different biological matrices, potentially contributing to differences in the results. This distinction underscores the versatility and practical advantage of our POC system in providing blood analysis. To compare both analytical methods, the Passing-Bablok regression was applied. It is represented in Fig. 6A with the interval of confidence (CI 95%). The corresponding values for the slope and Y-intercept are of 1 (0.9 to 1.0) and 2 (− 5 to 6) respectively. Since CIs includes the value 0 for the Y-intercept and the value 1 for the slope, it can be concluded that both methods measure the same concentration of the analyte without systematic differences and therefore that both methods are comparable in the concentration range evaluated [24]. A Bland–Altman plot for the methods comparison was also done because it allows the visualization of the agreement between two methods and any systematic bias or random error. It consists of the representation of the difference between the two measurements versus the average of the two measurements. The graphical representation can be seen in Fig. 6B. As can be observed from these results, there was a small positive bias (of 6.2 μM) when using the proposed automated POC, indicating a negligible systematic error. On the other hand, despite the high variability of errors between both methods, only 16 samples from 238 (less than 7% of the total) fell outside the CI according to the Bland–Altman plot. Indeed these 16 samples correspond to samples of a NH_4_^+^ concentration higher than 150 μM well above the threshold for hyperammonemia diagnosis. These results indicate that both the proposed automated POC and the reference method employed by the hospital are comparable, and the errors are randomly distributed, calculating a mean error of 4%. The paired t-test (t_calc_ = 1.87, t_tab_ = 1.98, t_calc_ < t_tab_) also supported the agreement and comparability of the two methods, as it showed that there were not significant differences between them.

## Conclusions

A prototype of an automated POC analyzer for blood samples was developed and validated. The equipment was installed in the laboratory of the HSJD for a 2-month evaluation under continuous use and validated through the analysis of real blood samples. Despite the challenges posed by the reference method, which exclusively measures NH_4_^+^ in plasma (rather than blood), as well as the difficulty in measuring NH_4_^+^ due to its significant increase in samples over time, excellent results were achieved. The comparison between the proposed automated POC and the current hospital method demonstrated that both methods are comparable in terms of analytical performance, confirming that the proposed POC system could be a reliable alternative for determining both healthy and pathological levels of NH_4_^+^ in blood samples, aimed at monitoring metabolic hereditary diseases and other conditions causing hyperammonemia episodes in clinics and hospitals.

Some modifications were made to the gas diffusion unit design compared to previous approaches, allowing for an extended unit lifetime. The modular configuration of the detection module enables individual unit replacement as needed, resulting in reduced maintenance costs.

This analytical system boasts unique characteristics not yet achieved by any other currently available system. Notably, it combines portability, reliability, ease of use, and affordability. Moreover, it can directly measure blood samples without prior treatment, significantly reducing overall analysis time. Full automation has been achieved, enabling the system to operate unsupervised with minimal intervention from laboratory personnel.

To create a commercially viable product, the setup should be further redesigned to reduce its dimensions and enhance portability. This would facilitate installation not only in hospital laboratories but also in emergency rooms, primary care centers, or even patients’ rooms, allowing for convenient bedside monitoring.

## Data Availability

Non-included data will be available from the authors by request.
